# Functionality Analysis of Electric Actuators in Renewable Energy Systems—A Review

**DOI:** 10.3390/s22114273

**Published:** 2022-06-03

**Authors:** Abhijeet Redekar, Dipankar Deb, Stepan Ozana

**Affiliations:** 1 Department of Electrical Engineering, Institute of Infrastructure Technology Research and Management (IITRAM), Ahmedabad 380026, India; abhijeet.redekar.21pe@iitram.ac.in; 2 Department of Cybernetics and Biomedical Engineering, Faculty of Electrical Engineering and Computer Science, VSB-Technical University of Ostrava, 17. listopadu 2172/15, 708 00 Ostrava, Czech Republic; stepan.ozana@vsb.cz

**Keywords:** electric actuators, linear actuators, rotary actuators, power consumption, spherical actuators, renewable system

## Abstract

Various mechanical, hydraulic, pneumatic, electrical, and hybrid actuators can alter motion per the requirements of particular applications. However, except for electrical ones, all actuators are restricted due to their size, complex auxiliary equipment, frequent need for maintenance, and sluggish environment in renewable applications. This brief review paper highlights some unique and significant research works on applying electrical actuators to renewable applications. Four renewable energy resources, i.e., solar, wind, bio-energy, and geothermal energy, are considered to review electric actuators applicable to renewable energy systems. This review analyses the types of actuators associated with the mentioned renewable application, their functioning, their motion type, present use, advantages, disadvantages, and operational problems. The information gathered in this paper may open up new ways of optimization opportunities and control challenges in electrical actuators, thereby making more efficient systems. Furthermore, some energy-efficient and cost-effective replacements of convectional actuators with new innovative ones are suggested. This work aims to benefit scientists and new entrants working on actuators in renewable energy systems.

## 1. Introduction

An actuator is a vital component of any physical system enabling movements by converting an energy source into another, primarily electrical, air, or hydraulic energy, into mechanical force [1,2] to modify the current system’s state. Some major applications include the automotive industry, furniture-ergonomics, automation, industrial machinery, maritime applications, the medical industry, and renewable energy systems. Various types of actuators are in use (as per the power sources used) such as electrical actuators [3], hydraulic actuators [4], pneumatic actuators [5], mechanical actuators, and a combination of these such as electro-hydraulic [6], electro-pneumatic [7], electro-mechanical [8], self driven thermo-mechanical [9], etc. An electric actuator creates a load movement or an action requiring a force such as clamping, using an electric motor to create the desired force, converting electricity into kinetic energy to automate valves, or damper actions using precise flow control [10]. Electric motors may work on AC or DC supplies depending on the requirements of the application, limit switches, brake mechanisms, resolvers, temperature sensors, etc. The desired force is generated from the motor’s torque capability and automates industrial valves, process plants, flow control, thermal power plants, irrigation systems, etc. [11].

Renewable energy plays a pivotal role in tackling climate change by reducing greenhouse gas emissions and fossil fuel use. Energy conversion systems driven by actuators help ensure optimal conversion while transforming renewable sources into valuable forms. Rotary to linear motion is achievable using a mechanical transmission unit, called linear motion actuators, which are better in accuracy than rotary actuators, compact in size, and easy to use in many applications such as solar tracking systems [12], where solar panels can track the sun’s apparent motion; solar furnace systems [13], where the motion required to control the sun-rays by moving shutters; solar heater systems [14], where sun rays are concentrated on oil pipes (called active area) placed over the parabolic solar panels; solar cleaning systems [15,16], where two motions are performed (viz., *x*-axis and *y*-axis motion) to clean the whole solar panel; small-scale wind systems [17], where actuators are used as protection devices in high wind speeds; wave systems [1,2,18,19], where actuators are used in the conversion of wave motion to linear and rotary motion for energy generation; and bio-energy systems [20,21,22,23], where actuators automate the operation of geologically distributed systems.

Energy optimization is achievable by increasing energy generation or reducing energy consumption. In solar panel cleaning, Williams et al. [15] use piezoceramic actuators for self-cleaning operation. The results show that dust moves away from high to low vibration velocity. The convectional solar panel’s mechanical cleaning structure is called a gantry structure [24] and is operated by two rotary actuators. Using telescopic actuators offers the possibility of reducing the use of one rotary actuator for reduced overall cost and energy consumption. In solar furnace systems [25], instead of rotary actuators by applying linear actuators, one may neglect the mechanical dead zone for decreased operation time due to the quick response increased testing quality. For solar tree applications, a 2DOF spherical actuator [26] is more beneficial than a linear actuator. There are two advantages: less space required and a mechanical latch arrangement which uses reduced power while moving. Such an appropriate selection of actuator ensures an optimal system operation.

**Figure 1:**
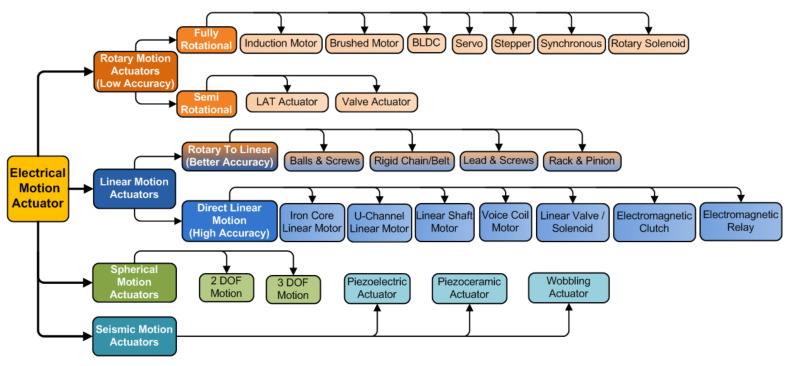
Active electric actuators according to their motion used in the renewable applications.

Actuators are mainly classifiable into active and passive actuators. The active actuators need an electric energy source for functioning. In contrast, passive actuators do not require a source and function based on natural energy, such as thermal expansion material, and energy stored in the spring [27]. Various required motions are circular, rotational, oscillatory (seismic), and rectilinear. Therefore, electric actuators are classified according to their motion, degree of freedom (DOF), and excitation sources for particular functional motion. As per motion, actuators are rotary actuators, linear actuators, and a new one called a spherical actuator with multiple degrees of freedom [26]. Linear motion actuators are split into two categories: rotary-to-linear (hybrid motion) actuators involving mechanical transmission units (also called electromagnetically linear actuators as shown in Figure 1) and direct linear motion ones not requiring mechanical transmissions units.

Actuators are used in renewable energy sources such as solar tracking applications to drive solar panels, solar dishes, heliostats, and solar cookers moving towards the sun throughout the day [28]. Solar water pumping applications use linear actuators in a water pump system [29], and solar tracking, or cooker, applications use actuators (motors or shape memory alloys) to drive the rotation of panels towards the sun throughout the day at a rate of 15${}^{\circ }$/h [27,30]. Depending on the temperature, certain materials in a thermo-mechanical actuator help control the collector’s angle of inclination to face the sun and provide increased production of about 39% compared to a fixed system. In addition, there are some bioenergy applications, where rotary actuators are used for agricultural machinery and the automation of geographically distributed biogas systems; portable wind energy applications [31] with actuator-controlled over-speeding; wave energy applications, where actuators are used in power-generation purposes [32]; smart grid or microgrid environment [33], where actuators control power generation and consumption; and geothermal power plants with electric valve actuators.

Rotary (also called electromechanical rotary) actuators help move large loads in angular increments. They include electrical motors with gearboxes used to change the direction of rotation or achieve the desired torque and speed with the desired adjustable rotation angle. Examples of the rotary actuators are induction motors, DC motors (brushed and brushless), synchronous motors, and special motors such as stepper and servo motors. Motors’ selection factors for actuation are speed, output torque, type of supply required, and motor duty cycle. However, induction motors, which give almost synchronous speed, are effective with high power requirements. Due to the low inertia, servo motor rotors working on AC or DC supply and controlled through pulse width modulation can quickly start and stop and are used to position mechanical parts precisely. Such precise motion control is needed in CNC machines and production assembly lines. In addition, DC servo, DC brushed, and BLDC motors are economical and less complex actuation devices. Unlike servos, a stepper motor that divides the rotary motion into equal small steps can provide 360-degree continuous rotation and is helpful for high torque output and high accuracy at low-cost [34]. However, due to electrical (cogging) and mechanical (dead zone) nonlinearities [35], these rotary actuators are relatively less efficient and have lower accuracy. Whenever we may face motor concussion problem [36], accuracy is improved by using special motors such as stepper motors and servo motors, which still face mechanical nonlinearities and, thus, low performance. In mechatronic systems, electric rotary actuators find extensive use.

A rotary solenoid is more suitable for small rotations and low torque applications than a costly and complex control circuitry stepper motor. It comprises an electromagnetic coil operated on a DC supply, rotor, and torsion spring. The change in supply polarity is easily handled in the direction of rotation, providing a meager angle rotational movement around a fixed axis (usually ⩽$\pm 360^{\circ }$). Typical rotary solenoids have bi-stable positions that do not require power to hold either end position, minimizing energy consumption. It can drive camera shutters and mirror deflections, fluid/gas valves, the actuation of indicating instruments, application areas such as the packaging industry, medical devices, apparatus construction, and food technology for interlocking purposes. Rotary-to-linear actuators (also called electro-mechanical linear actuators) comprises electric motors and mechanical transmission elements. The rotational motion converts into linear motion using mechanical transmission elements, such as the ball and screw mechanism, the lead screw mechanism, the rack and pinion mechanism, the belt and pulley mechanism, and rigid chains. Such elements slow down electric rotations, thereby increasing torque such that the load on the transmission part moves smoothly. The ball and screw are made out of a cylinder to allow the re-circulation of the balls, causing linear movement, but are used for distinct purposes and are not interchangeable [37].

Unlike rotary to linear ones, direct linear motion actuators have no transmission elements and provide high accuracy, resolution, and speed. There are various types, viz., iron core linear motors, U-channel linear motors, linear shaft motors, voice coil motors, linear motion solenoids, screw-type linear piezoelectric actuators [38], and electromagnetic clutches. The stator and forcer (called rotor), with a permanent magnet and electromagnetic coils, respectively, create linear force. However, the iron core brings cogging and eddy currents, heat, magnetic saturation, and lateral forces. U-channel direct linear motor eliminates such disadvantages, providing increased force through an additional permanent magnet on the opposite side for a double-sided configuration, with electromagnetic coils in an epoxy-mounted non-ferrous forcer aluminum plate. The absence of a magnetic core eliminates cogging, but reduces heat dissipation, and a double-sided configuration increases cost. Although they do not generate the same forces as an iron core motor and U-channel linear motors, they do not have to deal with the additional issues with that design. Voice coil actuators, working on the principle of Lorentz force, are a direct drive mechanism that allows for excellent fine-positioning over small movements.

An electromagnetic device called a “Linear Solenoid” turns electrical energy into a mechanical pushing or pulling force or motion for rapid ON and OFF operation and mainly comprises an electromagnetic coil, a plunger (armature), and a spring. When the coil energizes, the spring is compressed for the valve to open, and when the coil de-energizes, the spring moves downward to close the valve and control fluid/airflow as a solenoid valve. However, the disadvantages are that there are only two available positions, i.e., fully open or closed, generating a limited linear force for electrically opening doors and latches. Electric (also called electromagnetic) clutches have an electric actuation between two rotational gears (drive/input and driven/output) for mechanical transmission of torque through linear motion, i.e., operate electrically and transmit torque mechanically. The passage of a current through the clutch coil makes it an electromagnet producing magnetic lines of flux [39]. Although mainly used in biogas automation [20], clutches are also used in conveyor drives, packaging machinery, and factory automation. Apart from linear and rotary motion, the new spherical motion actuators [26] are multi-degree-of-freedom voice coil devices with a spherical-shaped stator, two coils, a ball-shaped rotor, and multiple permanent magnets (a plurality of magnets) [40] applied to robot eyes, joints, and solar trees.

This comprehensive literature survey focuses exclusively on renewable applications with electrical actuators. Particular actuators find a place in multiple renewable energy systems, while others have only specific or limited usage. Furthermore, there are certain advantages and disadvantages (provided subsequently in each Section) concerning actuator motion. We also suggest some alternative solutions for conventional actuators. However, the overall advantages of electric actuators against non-electrical actuators (such as hydraulic and pneumatic) are given as follows:

(i) Lower response time: Electrical actuators are directly driven without auxiliary accessories, unlike hydraulic actuators, which comprise motors, hydraulic tanks, controlling valve pipes, and cylinders, and hence are fast operating.
(ii) Lightweight: Compared to non-electrical actuators, electric actuators are lightweight and easy to install, with no adverse effect on system performance. The cost of overall installation and material is reduced [15,41].
(iii) Precision control: The low tolerances compared to non-electrical actuators such as gear backlash, slack, and inherent flex provide highly precise positioning [42].
(iv) Compact in size: Electric actuators are compact due to their direct electromagnetic energy conversion system, and they are designed for any space and physical size [26].
(v) Clean: Electrical actuators are a clean source of energy with no chance of leaking or dirty work areas [43].
(vi) IoT compatible: Motion and position are easily measurable due to inbuilt rotation sensors, and encoders and easily transfer to an IoT environment [44,45].

The paper’s structure is as follows. Section 2 reviews various types of electric actuators in different solar system applications such as solar tracking, furnace, heater, and solar panel cleaning. Section 3 presents a brief review of electric actuators used in wind applications, especially miniature rooftop wind turbines and wave energy applications. Then, Section 4 reviews actuators used in bioenergy applications, including agriculture and irrigation. Section 5 presents important performance parameters of actuators and evolution. Finally, Section 6 presents the conclusions and future direction.

## 2. Actuators for Solar System Applications

Solar energy is a free and abundant source available on this planet that is extracted in various ways, such as electric power generation using solar photovoltaic cells or solar-distributed collectors for heating applications, such as a solar water heater, furnace, heater, etc. Next, we present a survey of electrical actuators used in different solar energy applications.

### 2.1. Actuators for Solar Tracking Applications

**Figure 2:**
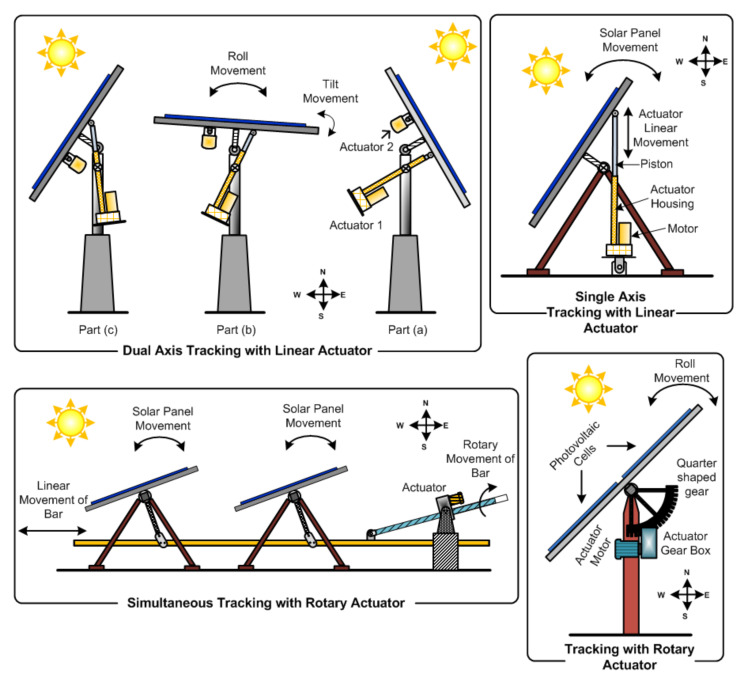
Electric actuators functioning in solar tracking applications.

Solar panels need to follow the sun’s movement to extract the maximum feasible energy. As the sun rises from the east and sets in the west, the tracker system aims to follow the sun’s movement or, during the day, keep the solar panel perpendicular to the sun, i.e., to follow the sun’s day to day and seasonal movement for maximum power extraction. The tracking system finds use in solar PV panels, in moving heliostat mirrors, parabolic concentrators, and solar collectors. We explore various actuators presently in use for solar tracking. Figure 2 shows individual solar panel tracking and simultaneous-tracking (i.e., group tracking) solar panels. The linear motion of the linear actuator is achieved from the motor’s rotary motion. Internally some mechanical motion change arrangements convert the motion, such as ball–screw, lead–screw, etc. This mechanical device also manipulates the torque and speed as required with an appropriate pitch of the screw.

Linear actuators and rotary actuators achieve solar tracking. Single-axis tracking, where a solar panel tracks the day-to-day movement of the sun (i.e., only east to west movement), is achievable only using one linear actuator [27]. The linear movement of the actuator is also shown through single-axis tracking with linear actuators in Figure 2. In the morning, the solar panel faces the east direction, and the actuators are in the initial position, i.e., actuator pistons are inside the actuator housing. (Note that the figure shows a west-facing panel and the actuator piston is entirely outside the housing.) As the sun’s movement starts, the actuator receives signals from the controller, and the motor starts rotating piecewise, which continues until sunset on the western side. At this time, the panel faces the west direction. At night, the solar panel moves back to the initial position through the linear actuator as the motor rotates anti-clockwise and the piston goes inside the housing. The sun changes its azimuth angle position as seasons change, and the panel cannot change the tilted position [12]. Hence, the panel cannot receive perpendicular sun rays, thereby decreasing the yield. When the PV panel is in the vertical position, the panel captures only a few rays and is therefore never used. When the PV panel is horizontal, the panel captures more rays. When moving along with the sun’s seasonal movement (i.e., tilt), the panel captures maximum sun rays, increasing the yield.

Moreover, single-axis tracking is achievable through rotary actuators, as shown in Figure 2 which provide roll movement achieved by electric motor gear arrangement. The rotary actuator has a speed reduction gearbox, which decreases speed and increases torque. Although rotary actuators are nothing but motors with a mechanical gear or a rack and pinion, they change the direction of motion with the desired torque. Interaction between the panel and actuator happens through a quarter-shaped gear disc attached to the solar panel base to achieve roll movement of the panel smoothly. This method is quite uneconomical for individual tracking due to the ample torque of rotary actuators. In contrast, such rotary actuators are suitable for simultaneous tracking where ample torque is required [46]. For receiving maximum sun rays, the solar panel should achieve both day-to-day and seasonal movements. So, we need dual-axis tracking achievable using two linear actuators named dual-axis tracking with linear actuators.

Three positions of the solar panel is shown in the dual-axis tracking of Figure 2, which pertain to the time of the day between 9:00 A.M. and 15:00 P.M. [27]. At 9:00 A.M., Actuator 1’s initial piston position is inside the housing, as shown in Part (a) in dual-axis tracking, with the panel oriented towards the morning sun. At noon, as shown in Part (b), Actuator 1’s piston position is partially outside of the cylindrical block panel oriented towards the afternoon sun. At 3.00 P.M., as shown in Part (c), Actuator 1’s piston position is outside the housing such that the panel is oriented directly under the mid-afternoon sun.

Actuator 2 helps account for seasonal changes. In a solar farm, individual panel tracking is non-economical. All solar panels in a particular row need to move simultaneously. Figure 2 also shows simultaneous tracking with rotary actuators. Only east-to-west tracking is possible with simultaneous tracking. Two actuators, used in a model by Hammed et al., is also called a two-axis tracker, where two independent motors (or actuators) ensure motion along an axis: one accounting for the azimuth angle and another for the altitude [47]. Jovanovic et al. use a linear actuator [48] to obtain the east-to-west motion of the solar panel. Weight reduction and cost-effectiveness are the primary considerations in this case and is accomplished by choosing a single linear actuator instead of the usual rotational actuator. Power consumption in the actuator depends on the anchor location. For example, for a moving panel of +45${}^{\circ }$, more consumption is noted than $-45^{\circ }$ rotation due to wind pressure during positive angle tracking. The ‘tilt’ and ‘roll’ (shown in Part (b) of dual-axis tracking in Figure 2) tracker employs a linear actuator and dampers [12]. Linear movement with a bar mechanism in the ‘roll’ axis finds use in the concentration of photovoltaic technologies, and two different heliostat-based tracking methods are discussed [49].

The tracking actuator operates in steps in intermittent sun-tracking systems, so the actuator power consumption is more. Ferdaus et al. describe an energy-efficient solar tracking system using a hybrid (continuous as well as static) dual-axis using two fully geared stepper motors for two different axes: 0.6 Watt power consumption in an actuator and a 25.62% energy gain recorded as compared to a static solar panel system [50]. In addition, Batayneh et al. [51] apply three-step intermittent tracking, with 0.4 Watt power consumption in actuator and an around 91–94% energy increment compared to continuous tracking. As we increase the intermittent step by optimization method, there is a saving in actuator power consumption, and almost the same gain is obtained compared to continuous tracking. Still, as per [52], the simulation results at one stage step by step tracking consume considerable power in the actuator because of the overshooting occurring when the motor is turned on/off. The energy consumption for achieving step-by-step motion laws is slightly higher than that for continuous motion. Table 1 shows results that after the fourth step, the actuator consumption is more than the continuous system, and the energy gain is also less.

Best performance through solar tracking is achievable when the tracker’s tilt angle synchronizes with the annual changes of the sun’s altitude (i.e., the sun’s seasonal motion). However, a single-axis tracker with an electric linear actuator sees higher energy consumption on sunny days and lower at smoggy times, that is, depending on the local weather conditions [53]. Elsewhere, Ponniran et al. evaluate a single-axis solar-tracking system’s performance for three different rotary actuator motors used individually (AC induction motor, DC motor, and stepper motor) [46] in comparison with linear actuators. Finally, in terms of economic considerations, Argeseanu et al. evaluate the cost factor in tracking systems by designing a low-cost dual-axis solar tracking structure with motor [54]. Sensor-based tracking is more accurate than azimuth-based tracking [55].

There is a significant benefit of dual-axis tracking [56]. Without tracking, the yield graph looks like a half-sine wave with the peak at noon. With dual-axis tracking, the yield is a trapezoidal shape of a larger area, but there is a need for two actuators, both in the case of linear and rotary actuators. Such actuators with dual-axis tracking are effective in solar farms with several rows of panels placed. The number of actuators required is less in such cases due to simultaneous tracking, causing a significant uptick in power generation. However, such actuators are not suited in the solar tree concept where space is an issue. Two actuators for each panel in dual-axis tracking are needed, group tracking is not feasible, and the number of actuators required is more like a solar street light system.

**Figure 3:**
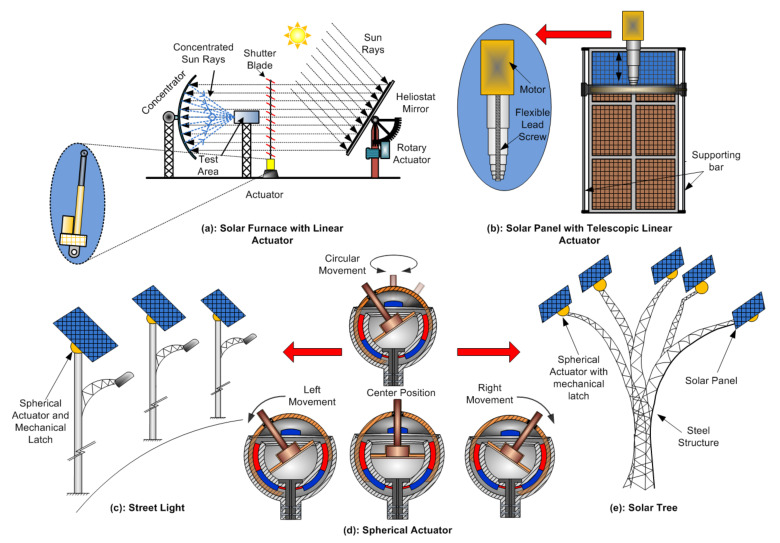
Proposed alternative actuators view: (**a**) solar furnace with linear actuator, (**b**) solar panel with telescopic linear actuator, (**c**) street light application with spherical actuator, (**d**) 2 DOF spherical actuator, (**e**) solar tree application with spherical actuator.

The advantage of a solar tracking system with one actuator is initial cost reduction, but it decreases yield. In contrast, solar tracking with two actuators increases yield but also the initial cost of the system. To only seek the advantages of both, a new type of actuator called a spherical actuator [26] may be an ideal replacement of the linear actuators in applications such as a solar tree, such that it accomplishes the task of the existing dual-axis tracking with a single spherical actuator. Furthermore, two degrees of freedom (DOF) spherical actuators shown in Figure 3d with mechanical latch can perform tracking on both axes for increased energy extraction and reduced cost. In addition, due to the latching arrangement, the energy is required for the actuator only when motion is required; hence, it can be more energy-efficient than two-axis tracking. The most significant advantage of the spherical actuator is its compactness; it can be used in solar tree application as shown in Figure 3e and street light applications as shown in Figure 3c.

### 2.2. Actuators for Solar Furnace Applications

**Figure 4:**
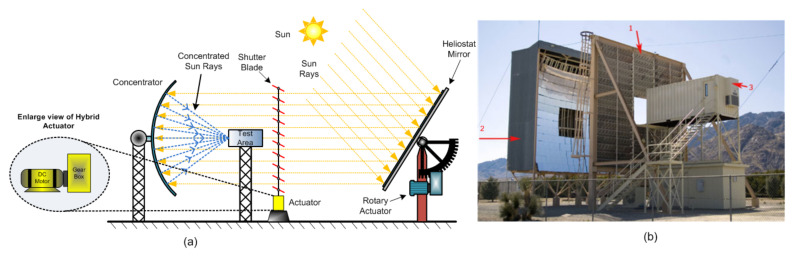
Rotary actuators functioning in solar furnace application (**a**) Schematic Diagram, (**b**) U.S. Army’s white sands solar furnace commissioned in 1972 [57,58].

A solar furnace is a renewable (green) source of energy. It is a thermodynamic structure [13] that provides concentrated solar radiation, as shown in Figure 4a, using concentrated solar power to produce very clean and high-temperature heat, around 3500 °C (6330 °F) [25].

A solar thermal power generation plant commonly employs a solar furnace system different from a solar energy system because it is an indirect use of sunlight reflected and then concentrated on one point of interest. The generated heat is mainly valuable for industrial applications, the heating process of a substance, material testing, charging experiments, melting steel, hydrogen fuel, and foundry applications. The primary solar furnace structure with components such as heliostat mirrors (Not visible in Figure 4b, but shown in Figure 4a), a parabolic concentrator (Number 2, shown in Figure 4b), and mechanical shutters (Number 1, shown in Figure 4b) operated through an electric actuator is standard for all applications. A flat heliostat reflects the solar radiation coming from the sun towards parabolic concentrator mirrors. Rotary actuators or linear actuators placed at the heliostat mirrors track the sun’s movement to reflect maximum sunlight, passing through rows of horizontal mounted mechanical shutters whose rotary motion controls and regulates the reflected sun rays as per the heat requirement [59]. The general opening and closing range of shutters are 0–90${}^{\circ }$, where $0^{\circ }$ means shutters are fully open to allow passage of maximum sun rays coming from flat heliostat, and at $90^{\circ }$, the shutters fully close to prevent means sun rays from passing to the concentrator. Rotary and linear actuators achieve such type of motion, but primarily, rotary actuators find preference due to large torque requirements. Generally, AC or DC motors and gearboxes help change the position of the shutters [13]. Finally, the sun rays reaching the parabolic mirrors are concentrated onto a test area (Number 3, shown in Figure 4b) for testing electrical, mechanical, and metallurgical materials under high-temperature heat. Specifically, molten salt is pumped into the furnace test area (also called the tower) in a solar thermal power plant. The molten salt temperature rises from around 300 ∘C to over 600 ∘C. Then, high-temperature molten salt is transported to a storage tank and is used for steam generation and eventually electric power generation.

The disadvantage of a rotary actuator in moving shutters is that there will be no quick response of opening and closing shutters due to the backlash nonlinearity of the gearing box. In addition, there is the additional disadvantage of increased space requirement. Instead of a rotary actuator, a low-power linear actuator will be advantageous, as shown in Figure 3a, because there will not be an active load on the shutters, and so the linear actuators perform the shutter’s task with low energy consumption and also occupy less space. Adjustment of shutter angle with a rotary actuator enables control of solar flux by controlling the speed of shutter opening, and closing [60]. An aluminum shutter blade operated by a closed-loop serve mechanism helps control sun rays passing from solar mirror to concentrator. Costa et al. also use a solar furnace [13] wherein brushless DC motor and gear mechanism, i.e., rotary actuator, controls the shutter blade movement. Shutters control solar radiation, which helps reduce the concentration so that overheating of the solar furnace is prevented.

For instance, a 1 MW large solar furnace that has been operational since 1968 in Font Romeu Odeillo [43] has seen an upgrade from hydraulic actuators to electric brushless DC motors for efficient operation. These revolutions in the application of the actuator and their power consumption are shown in Table 2.

The first generation saw the use of hydraulic actuators, but hydraulic leaks and actuator pressure losses harm the effectiveness of solar tracking and cause heliostat oscillations causing rotation axis wear. Therefore, an electric actuator is used in the second generation due to efficient and reliable tracking control. Stepper motor and BLDC motor with ball screw jack obtained linear motion. BLDC has less power consumption than the stepper motor and requires less maintenance.

### 2.3. Actuators Used for Solar Heating Applications

**Figure 5:**
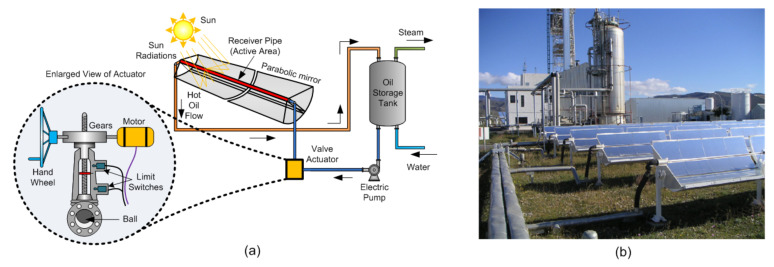
Valve actuator for distributed solar collector field: (**a**) Schematic of plant, (**b**) the ACUREX parabolic-trough-distributed solar field collector [62,63].

In the crisis of 1973, oil prices rose dramatically, resulting in the search for sustainable solar heat energy sources for electricity or industrial purposes [14]. Two types of solar heat energy collector mechanisms: (i) a receiver pipe type (active area) and (ii) parabolic trough-based distributed collector system [61], are explored. It is helpful in steam generation for thermal plants, industrial processes, desalination plants, etc. An example of such a plant is the ACUREX parabolic-trough distributed solar field collector located in the Tabernas Desert (Southern Spain), as shown in Figure 5b and their schematic in (a). Trough collector systems comprises a parabolic mirror used to focus sunlight on the active area of the pipe for the oil to absorb thermal energy along the focal line. The system also comprises a heat transfer fluid (HTF) (typically synthetic oil or water), an oil storage tank, a heat exchanger, a electric control valve actuator, an electric pump, etc.

The oil pumped from a storage tank (see at the right in Figure 5) passes (at ambient oil temperature) through the electric valve actuator to the field to be heated by concentrated solar radiation and returns to the tank via injection at the top of the storage tank. Once the heat energy is extracted from hot oil, the cold oil is re-injected at the bottom of the storage tank [64]. Suitably adjusting the oil flow rate helps to regulate the outlet oil flow temperature and track a desired temperature reference value using an electric valve actuator (along with the control system) that plays a vital role in temperature maintenance [61]. Electric valves comprise an electric motor, gear mechanism, hand wheel, limit switch, and butterfly valve working at three positions: two end positions and an intermediate one. One of the extreme positions is perpendicular to the oil flow, i.e., valve closed position, the other is parallel to oil flow, i.e., fully open position, and the third (intermediate) position where continuously and smoothly rotates the valve to regulate the oil flow at any flow rate.

To understand the operation of the valve, consider an initial closed position. When the motor starts, it rotates the gear mechanism and changes the direction of rotation. The gear mechanism rotates the vertical shaft at a slow rotation, causing the torque to increase and speed to reduce. The butterfly valve attached to the vertical shaft changes position according to the direction of motor rotation. The oil flow rate is proportional to the valve position. Two limit switches at the upper and lower end operate through a small lever with a vertical shaft for valve automation and protection. When the motor and vertical shaft rotation is clockwise, the valve changes position from closed to open. When fully open, the upper limit switch operates through the lever, then the motor stops rotating. In the anti-clockwise direction of rotation, the valve changes position from open to close. When fully closed, the lower limit switch operates such that the motor stops rotating.

An electric valve actuator is sound in operational control and helpful in protecting from stagnation and overheating. Frank et al. [65] explain the problem associated with the industrial solar heat processing system and different measures to prevent them, stating that weekend shutdowns or company holidays raise the stagnation problem and technical malfunction or a power outage causes overheating. Both problems increase pressure, resulting in the opening of the safety valve. Partial emptying heat transfer fluid (liquid or vapor), with loss of heat, cause additional costs and maintenance. Some passive and active strategies help control stagnation and expansion. In the case of active control strategies, pump-electric valve actuators and appropriate rotary or linear actuators are in use. For handling the stagnation pump-valve system, evacuated tube collectors, and overheating prevention active cooler loop, automatic defocusing of the mirrors is useful. Motlagh et al. propose that pneumatic actuators are helpful to control valves in the energy sector, such as in power plants and the oil and gas industries [66]. The use of electric control-valve actuator technology allows for energy efficiency. Linear actuators control passive structures such as shutters in solar air heaters applications, which are helpful for regulated room heating and reduced electricity bills [67].

### 2.4. Actuators Used for Solar Panel Cleaning Applications

**Figure 6:**
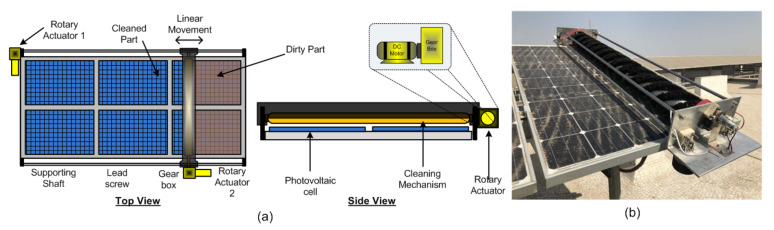
Actuators for solar panel cleaning: (**a**) Hybrid actuators functioning in solar cleaning application, (**b**) prototype model of wheeled automatic cleaning robot [24].

**Figure 7:**
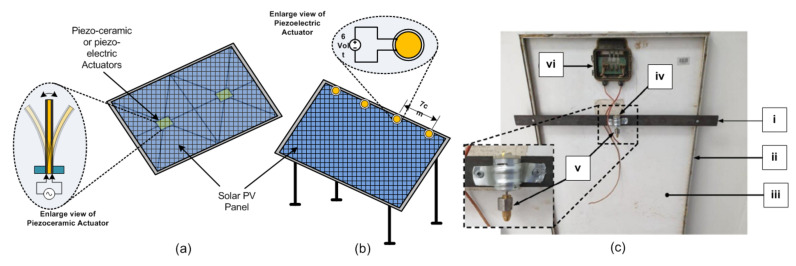
Vibration-based solar panel cleaning applications: (**a**) Piezo-electric actuator located on the back side of the panel; (**b**) Piezo-electric actuator located on the surface of the panel; (**c**) Eccentric Rotating Mass (ERM) motor actuator located on the back side of the solar panel, where (i) iron base for motor, (ii) solar panel frame, (iii) back side of the panel, (iv) ERM motor, (v) ERM, and (vi) connection box [70].

**Figure 8:**
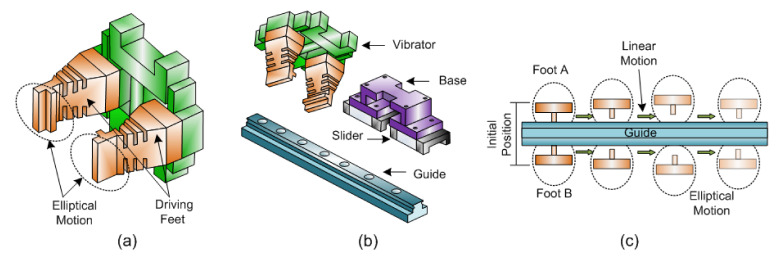
Linear piezo-actuators functioning in solar cleaning applications; (**a**) elliptical motion of their feet, (**b**) the driving bar and the support, and (**c**) the elliptical motion and linear motion for both the driving feet [41].

The performance of solar panel power generation depends on the quantum of sunlight. However, bird poop, dirt (or dust), or pollen on the solar panels prevents light from entering the solar cells, thereby affecting the efficiency of the solar cell, leading to reduced energy production. Maximum solar energy extraction is possible with regular maintenance and cleaning of the solar panels. Using a good cleaning technique recovers up to 95% of the solar cells’ power-generating capacity [15]. This section discusses the actuators used for effective cleaning methods specifically include electrical actuators such as electro-mechanical actuators (Figure 6), piezoceramic actuators (Figure 7a), piezoelectric buzzer (Figure 7a), piezoelectric actuator (Figure 7b), and linear-piezoelectric actuator (Figure 8) mechanism.

Deb et al. demonstrate a water-free automated cleaning service unit, comprising two DC geared motors, a lead screw, supporting shaft, and timing belt drive mechanism, and the cleaning task is completed using a rolling soft cleaning brush as shown in Figure 6a [24]. These are hybrid actuators that provide rotary-to-linear motion through suitable mechanical transmission arrangements. However, the problem with such mechanisms is that the movable cleaner can become stuck at one side due to there only being one side drive, and so there may be chances of interrupted motion. Moreover, due to the nonlinearity present in the mechanical transmission unit, the performance of the moving system reduces with time.

The solution to this issue is that instead of two rotary actuators, one may use only one actuator called a telescopic actuator, as shown in Figure 3b to push and pull at the center of the cleaner for smooth and symmetric motion. This reduction in an actuator also reduces system nonlinearities, making the system less energy-demanding. Furthermore, such a structure is helpful for solar farms due to its autonomous working. Wheeled semi-automatic cleaning robots having two geared motor actuators connected to two ends of the robot also solve the motion interruption problem [24]. Panat et al. [68] shows the electrostatic dust-removal system with a linear guide slide stage actuator motorized by a stepper motor. Williams et al. [15] present an array of nine piezoceramic actuators attached to the backside of the solar panel surface to handle the dust falling on PV panels by mechanical vibrations (acceleration). Nine actuators are excited by three three-channel piezo amplifiers. Sine sweep excitation of different magnitudes and frequencies (especially significantly higher frequency from 400 Hz to 5000 Hz) shows that high acceleration, i.e., the best dust cleaning motion, efficiently improves the dust-removal process. Such cleaning methods are effective in Martian surface missions or lunar surface environments. Dawson et al. [69] state that instead of piezoelectric actuators, inexpensive and low-power piezoelectric buzzers are helpful for panel cleaning. Incorporate piezoelectric vibrational actuators into the structural supports of photovoltaic panels to occasionally induce vibrations to loosen accumulated dust and are mounted at nodes of a grid of spars, as shown in Figure 7a. Despite being compact, low-power consuming, and inexpensive, the commercially available buzzers, which typically have operating frequencies in kHz, need a redesign for lower photovoltaic panels with lower natural vibrational frequencies.

Alagoz et al. [16] demonstrate that the acoustic surface wave (SAW) vibrations, gravitational forces, and panel surface slope play a significant influence on soiling mitigation. Figure 7b shows four piezoelectric actuators attached at the top surface of the panel driven by a pulse generator, generating pulse-type vibrations. Piezoelectric actuator spaced 7 cm apart and working at the resonant frequency. The traveling pulse strikes the surface dust particles, causing acceleration, leading to dust migration from the panel surface. Due to gravity, experiments have shown that bigger particles can easily slide down and away from the panel surface. In addition, a piezoelectric-actuator-based panel cleaning wiper arrangement are used [71,72]. The three actuators, viz., a piezoceramic actuator, a piezoelectric actuator, and a piezoelectric buzzer, are lightweight, so there is no adverse effect on the tracking system. Other important benefits of such actuators are its high torque-to-volume ratio and there being no electromagnetic interference in their operation. However, due to significant vibrations, the solar cell may deteriorate, which poses a research problem of optimization of the location of the piezoelectric actuators. Al et al. [70] generate vibration with motor and Eccentric Rotating Mass (ERM). Such wobbling motion will help to migrate dust particles down as the panel is inclined, as shown in Figure 7c. Lu et al. [41] proposed a prototype of a linear-piezoelectric actuator with an elliptical motion of their feet shown in Figure 8a. It consists of two vibrator plates of piezoelectric material PZT-8H, each plate of size 10 mm × 10 mm × 1 mm. The electrodes and piezoelectric (plates stack not shown here) are bunched between the driving bar and the support as shown in Figure 8b. The application of a sinusoidal signal causes an elliptical motion as well as a linear motion for both the driving feet shown in Figure 8c.

The entire excitation structure being symmetrical, the actuator moves linearly along the guide fixed over the frame to cover the whole panel area with the attached wiper actuator’s base to be driven to aid in the cleaning operation. The advantages of such actuators are five times higher torque-to-volume ratio than electromechanical actuators, smooth operation, high holding position, and lightweight and flexible structure. Examination reveals that energy gain at effective panel width is more significant because L=260 mm >130 mm. That is, wiping a solar panel in the direction with a shorter wiping distance (or stroke) results in a better energy gain for a given active area. In addition, the speed of cleaning depends on the way of cleaning path. Panel cleaning is also performed with blowing technology. King et al. [73] demonstrated compressed air cleaning with a motor and compressor set. Synthetic jet actuators known for injecting air jet at low power consumption can replace said blowing method for solar panel cleaning application [74,75].

Piezoelectric material creates vibration and also generates electrical energy against vibrations. Hamlehdar et al. [76] review energy harvesting methods by vibration-based techniques and focus on piezoelectric devices in energy harvesting from fluid flow motion. Piezoelectric actuators play a significant role in converting ambient energy into electricity for small and remote devices. In the wind-flow technique, energy harnessing generates electricity through piezoelectric methods such as flutter-induced vibrations (FIV), vortex-induced vibrations (VIV), and galloping. In the water flow technique, a cantilevered transducer having a piezoelectric actuator experiences the vibration due to the converging–diverging channel of water flow. The natural potential of fluid vibration available in ocean waves provides energy density 4–30 times higher than wind energy. This technique uses a piezoelectric beam coupled to the floated buoy to generate electrical energy against wave vibration. Among the various energy-harvesting methods, such as electrostatic, electromagnetic, piezoelectric, and hybrid methods, the vibration-based method has several advantages over other solutions, including high-density power output, high output voltage, and intrinsic cross-conversion capabilities, etc. Han et al. [77] measured the generated power by a PZT-based piezoelectric ceramic actuator, focusing on the deformation that affects power generation. Deformation applied in the size of the piezoelectric ceramic, depth of compression, and speed of compression contribute to the deformation of a single PZT-based piezoelectric element. Nechibvute et al. [78] present a concise review of power generation by piezoelectric microgenerators and nanogenerators for wireless devices such as sensors, microelectromechanical systems, etc. Battery-operated wireless sensor network performance depends on batteries’ capacity and needs replacing when discharged. Hazardous sensing environments do not allow for frequent battery replacement, so in such situations, piezoelectric-based continuous generation makes the sensing device autonomous. In addition, such actuators play an essential role in low-power remote sensing devices for optimizing the structure’s weight. The actuator’s power consumption and net improvement in system power is analyzed and summarized in Table 3. Actuators play a significant role in solar tracking and cleaning, and the efficient use of electric actuators improves system efficiency.

## 3. Actuators in Wind Applications

**Figure 9:**
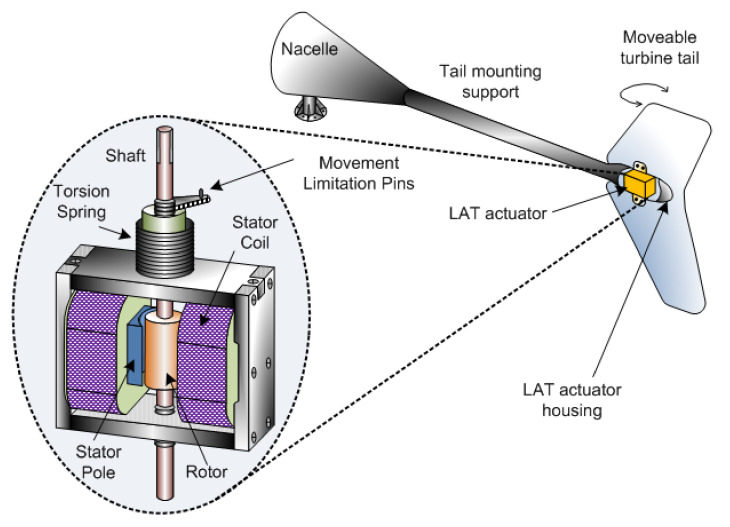
Limited-angle torque actuator (LAT) [42].

A relatively low wind speed (<5 m/s) makes the deployment of conventional wind turbines in residential areas non-productive. Therefore, new topologies of generators are needed to operate favorably in such low wind speed regimes for adequate energy capture [17]. Usually, wind-direction-oriented turbines are more efficient vis-à-vis turbines operating in winds from any direction. In addition, such small wind turbines favorably orient with a rear rudder as an effective brake for damage mitigation at high speeds. In addition, two actuators protect from higher speeds beyond a threshold voltage via unipolar control—the first is a limited-angle torque (LAT) actuator (called ON-OFF bipolar actuator) used on a 2.5 kW wind turbine with a nominal wind speed of 500 RPM (shown in Figure 9). The second is a stepper motor actuator with a reduction gear (i.e., rotary actuator) [82]. The actuator comprises a permanent magnet rotor mounted on two bearings—the rotor movement is limited by two dead-end pins mounted on one of the steel plates. Stator poles (with two windings connected in series) mounted on supports of the same steel type fixed to the plates’ ends together form a rigid assembly.

The nominal coil operating voltage is $V_{nom}=200$ V, and the threshold voltage is $V_{min}=190$ V and $V_{max}=210$ V. The comparator circuit measures and compares the input voltage to preset values, e.g., $V_{min}$ = 190 V and $V_{max}$ = 210 V. Because the actuator does not take action between these voltage levels, the system restricts the LAT oscillation at high speed using two comparator values. On reaching the threshold voltage (high generator speed), the rotor moves between two stationary locations, furling the wind turbine. A circular torsion spring placed on the rotor shaft causes the return of the rotor to the initial position after the wind turbine deceleration. The nacelle and tail align with the wind direction for wind speeds below a certain level. As shown in Figure 9, the LAT actuator mounted on a tail is activated when the voltage exceeds the threshold, causing the tail to rotate perpendicular to the rest of the wind turbine [42]. With the nacelle no longer aligned in the wind direction, the blades are not driven efficiently, ensuring speed reduction. Upon the restoration of regular wind speeds, when the windings receive a reverse polarity voltage, the actuator rotor returns from $90^{\circ }$ to $0^{\circ }$. The detection levels are adjusted to accommodate different applications and dynamics. The angular displacement solenoid actuators [83] using springs provide torque at a specific desired position despite no electrical current passing through the motor. A solenoid actuator is used as an ON–OFF bipolar actuator in place of LAT. A similar controlling operation has been done with the stepper motor-based actuator. Instead of two coils as LAT, two different comparison and control circuits and one angular moment element play an important role. Table 4 shows a comparison of actuators based on selected parameters. Such actuators are restricted to the system within rated power.

**Figure 10:**
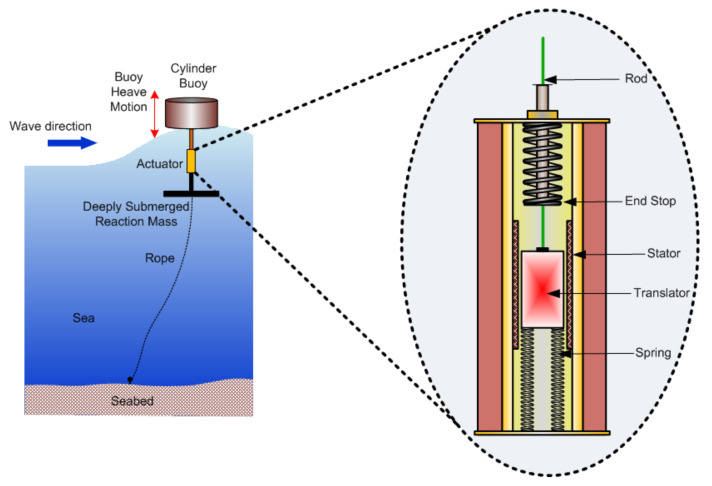
Cylindrical buoy actuator for wave applications.

Wave power is a predictable renewable energy source that can create more electricity than wind and solar combined. Wave energy absorption from ocean waves varying in time scales and produced by wind action indirectly from solar energy [1] is a hydrodynamic process resulting in radiation wave phenomena. However, the conversion of wave kinetic energy into smooth electric energy is difficult due to the low frequency of wave motion (~0.1 Hz), random wave, and high-force oscillations [84]. A “smart” oscillating-body device, also called a heaving buoy, that travels freely up and down with the rise and fall of the waves converts wave motion into linear motion for the power-generation system [18]. Linear motion is created by reacting an oscillating buoy against a fixed reference frame called deeply submerged reaction mass, as shown in Figure 10, and it is stationary due to its heavy mass.

The reaction mass connected to the seabed through the rope ensures a fixed floated position in the sea. A linear actuator placed between the floated buoy and submerged reaction mass transfers the relative motion of these two through a rod connected between translator and floated buoy. A device called power takeoff (PTO) helps the rod’s linear motion (up and down) to convert into usable power [19]. A piston pump device (such as a hydraulic drive or mechanical drive), air or water turbines, rotating electrical generator, or linear electrical generator can serve as a power takeoff device (PTO) [2]. The piston pump supplies high-pressure water to a hydraulic turbine [1], where linear motion converts into rotating motion through water turbines placed onshore. When a rotating electrical generator is available as a PTO, the actuator first converts the linear motion into rotational motion so that the rotating generator generates electricity. For offshore systems, the rotating generator is in the PTO itself. Figure 10 shows the enlarged view of the cross-section of a linear generator that generates electrical power through linear actuation. It has two dead-end stop springs and stator electromagnetic coils placed in the peripheral of the cylindrical body of the actuator. A translator with a solid permanent magnet providing magnetic flux inside the generator can freely move if vertically placed at the center. The relative motion of the buoy and the reaction mass is directly transferred through the rod to the translator for electricity generation through linear motion and the principle of electromagnetic induction.

Actuators used in wave energy conversion (WEC) systems are categorized into oscillating water columns (OWCs) and oscillating body systems or wave-activated body systems. Figure 10 shows the second category along with a linear PTO mechanism. This type of motion conversion system (actuator) and PTO device is essential in the perspective of wave power conversion efficiency. Aderinto et al. [85] explained detailed PTO types, scales, and efficiencies, among which were the efficiencies of five different combinations of actuators and PTO devices, and the results of their numerical simulation with computational fluid dynamics (CFD) are shown in Table 5. The direct-drive linear generator system with an oscillating buoy shows 95% efficiency, which is the highest compared to others. Except for linear generators, all other PTOs have losses in their motion conversion process, but in linear generators, no motion conversion is required and, therefore, comes with higher efficiency.

Rahman et al. [86] reported that PTOs, which are also built of piezoelectric actuators, are used to produce a significant quantity of electrical power from the ocean. Therefore, energy converters can be used to power applications that function in the ocean, such as various electrical sensing elements, floating harbors, robotics, etc. Wave energy converters (WECs) can improve their energy conversion efficiency by including several types of piezoelectric materials. Experiments reveal that by straining the material with a pressure of 1.196 kPa at a frequency of 20 Hz, 0.2 W electric power with 2.2 V may be obtained using this technology.

Next, we discuss actuators used in bio energy-related systems such as agriculture, solar irrigation, biogas, biomass, etc.

## 4. Actuators in Bioenergy Applications

Huo et al. present a distributed control system and communication topology for safe and automatic (start/stop) regulation of biogas driven appliances such as a rice cooker, stove, and water heater, etc., configured with a ZigBee endpoint to control the relevant actuators and transmit the data [20]. For example, a biogas rice cooker typically consists of cooking and warming switches for a three-stage operation: (1) heating with both switches pressed; (2) warm stage with cook switch lifted through a step-motor if cooking is over; and (3) the turn-off stage, with both switches lifted. The step-motor rotates at a fixed angle as commanded to press or elevate the switch. A single standard biogas stove with switches and gears controlled by a Knob’s rotation angle comes with a step-motor, an ignition circuit, and an electromagnetic clutch to turn the knob on when the rotational power of the step-motor passes through the clutch to the gear and manually when the clutch is out of power. The mechanical structure of the biogas water heater is similar to the rice cooker. Biomass (grate) combustion being a reasonable alternative to fossil fuels, it is expected to be a significant player in a sustainable future [88] and can fire a wide range of fuels of varying moisture contents, requiring less fuel preparation and handling [89].

**Figure 11:**
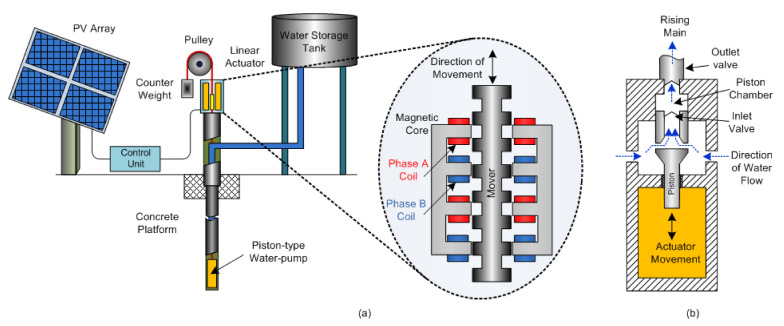
Linear water pump actuators (**a**) variable-reluctance linear stepper motor; (**b**) linear pump [29].

Agriculture wastes (most common are wood chips and sugarcane bagasse) are the primary fuel source for biomass furnaces. These biomass grates require actuators to operate an intermittent ash removal system. Biomass-fired power plants are cogeneration facilities producing heat for process heating and electricity generation, driven by electric valve actuators for feedwater treatment, steam generation, turbine injection, flue gas cleaning, and process heating. In addition, electromagnetic actuator systems robustly driven by an induction servo motor drive (rotary actuator) are used in agricultural applications [21]. Sontake et al. [90] present a review of solar PV powered water pumping system with a linear actuator as shown in Figure 11a. The system comprises a piston-type pump, a two-phase variable-reluctance linear stepper motor that works as a linear actuator, a reservoir, a pulley, and a counterweight. The enlarged view of the linear actuator provides the linear vertical motion to the piston-type pump. The solar PV array provides power supply to the control unit, converting it to a two-phase supply using converters and fed to the linear stepper motor. Due to the two-phase supply, electromagnetic coils A and B energized alternately to create downward linear motion.

For creating an upward motion, the controller changes the sequence of the excitation supply of the electromagnetic coil. Still, there is a need for a more significant up-stroke to lift water, so a counterweight is helpful. Aliyu et al. [29] present an optimized design for a solar water pumping system, as shown in Figure 11b, which shows a cross-section view of the cylindrical actuator, comprising an electromagnetic coil, a piston, a piston chamber, an inlet valve, an outlet valve, and a piston return spring, which provide restoring force (not shown). The inlet valve is submerged in the water by a water tank. The electromagnetic coil excited by the PV panel supply sets up a solid magnetic attractive force, causing the piston to move upwards. With the coil excitation removed, the piston returns due to the restoring force of a spring. The piston’s vertical linear (up–down) movement happens due to the magnetic field and the return-spring. On the upward stroke of the piston, water in the inlet and piston chamber is expelled towards the outlet valve into the rising main. The piston drops downward at the end of the up-stroke, closes the outlet valve, and fills water into the piston chamber via the inlet valve. Due to the non-returning valve arrangements, water flow never returns once it enters the piston chamber, providing an efficient water pumping arrangement.

**Figure 12:**
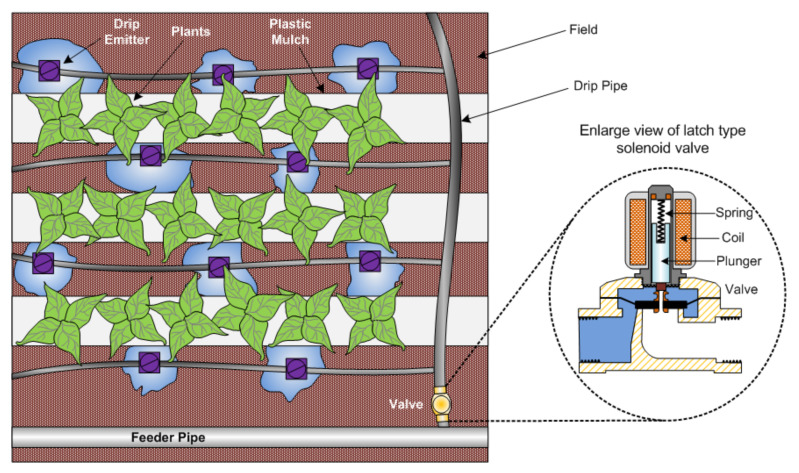
Localized irrigation system and latch-type Solenoids [93].

Talavera et al. discussed agro-industrial and environmental fields where remote actuator devices such as valves, pumps, humidifiers to maintain consistent humidity, relay, and alarms, among others, are in use [22] to optimize the usage of water by scheduling irrigation timings and proportions, fertilizers, and pesticides as per the state of the crop, the growth cycle, and based on available information from weather prediction systems or an on-site wireless network of sensors and actuators over an IoT platform to control the state of the process for irrigation, fertilizers, pesticides, illumination, and access control. Elsewhere, Yoo et al. describe an automated agriculture system based on an interconnected actuator network for monitoring the growth of greenhouses plants, which depend on ambient light, temperature, and humidity, through controlled illumination via a relay actuator [91]. If an agricultural intrusion is detected, the proposed system-generates alarms in the farmer’s house [92]. Localized (drip or trickle) irrigation applies water at low pressure (<2 bar) to a small area of the soil surface, sometimes only part of the root zone, and is widely adopted in greenhouse vegetables [93], through hardware components such as pumps, valve actuators, sensors, pipes, tubing, and emitters. A scheme of a localized irrigation system with two-way latch-type solenoid valves with limited stroke length, two ports of water flow (inlet and outlet), and two switching states (open or closed) to start/stop the controlled water flow, which is usually in open condition so that the valve closes on power failure [23,94], is shown in Figure 12.

Generally, latch-type actuators operate on a direct current (DC) supply. The selection criteria for such actuators depend on the pipe connection size, operating voltage, seal material, pressure, temperature, bore size, and more. When the coil receives supply, it becomes energized, and the movable plunger changes its state (ON or OFF).

There are many other renewable energy sources not covered in this manuscript that also deploy electric actuators. For example, the direct utilization of geothermal heat energy extracted in the form of liquid or air using a ground heat pump system and utilized as warm or hot water (in the winter season) and vice versa in the summer season, which is clean and safe, is also helpful for electricity generation and mineral production [95]. The water circulating loops of the ground heat pump system consists of centrifugal pumps and flow controlling actuators [96].

## 5. Important Performance Parameters of Actuators and Evolution

Some major performance parameters, their specifications, and applications are shown in Table 6, which gives a summary of the actuators’ major parameters/specifications. Some of the papers do not mention specifications, so only the parameters are provided. The actuators’ drive range determines the size of the feasible solar tracking region [49] (i.e., stroke length). In addition, the maximum rated load, maximum speed, continuous power, and system backlash are all vital performance criteria to consider when selecting linear actuators [97].

A motor, gears, and switches make up an electric rotary actuator. In some applications, the motor speed is an important parameter, e.g., the minimum shutter traveling time is required to operate mechanical shutters of the solar furnace system. There is no significant external load on shutters except for its weight. Some applications require more significant torque than the speed of the operation: in solar tracking applications, the solar panel weight is there; in a biomass grate boiler system, a rotary valve operates against fluid pressure. In such cases, the actuator gear ratio plays a significant role. A gear train is used in electric actuators to increase motor torque and control the actuator’s output speed. Therefore, the actuator torque and range of motion should be considered while choosing an electric rotary actuator. In computerized numerical control machines where precise motion control is essential, servo motors are suitable due to low inertia, enabling quick start and stop options. In addition, a servo amplifier can modify torque and speed effortlessly. Figure 5 has already shown a schematic diagram of an electric valve actuator that can control water circulation rate. However, some advantages, disadvantages, and their limitations are given in Table 7.

Overheating protection is available to prevent major breakdowns. Piezoelectric actuators are a new class of actuators for active mechanical system control. Piezoelectric materials have been adapted to applications that require just small quantities of displacement, such as solar panel dust cleaning, despite the magnitudes of piezoelectric voltages, motions, or forces being modest and often requiring amplification. Therefore, the displacement, pressure, resonant frequency, and voltage applied to piezoelectric actuators are vital characteristics. Limited-angle torque actuators are especially used to protect small windmills against over-speeding. They work on a predefined voltage rating of the coil called the threshold actuator coil voltage. Therefore, the voltage and rotation angles are essential.

The electric actuator, initially only a motor with a set of gears and some mechanical torque limit switches enclosed in a box, was later coupled with some auxiliary components such as position feedback switches and over-temperature (thermostat) and over-speed protection, heater, and breather control. After that, electric actuator design improved in various hazardous locations such as oil fields, chemical plants, or gas pipelines, etc. The development of non-intrusive setups combining new and more effective electronic controls with communication protocols was the next step in increasing commissioning capabilities [98]. Wireless actuators are simple to use, implement, and commission these days. They provide both communication and diagnostics while obviating the need for costly wiring and installation [99]. Pneumatic actuators have long been used to control valves in power plants. The use of electric control-valve actuator technology allows for energy efficiency [66]. There are different actuators designed for the power sector, but electric actuators provide solutions for reducing energy waste in renewable energy-producing systems efficiently [100]. Next-generation actuators will develop with new wireless communication technologies such as the Internet of Things (IoT), 5G, and intelligent robots, which are also expected to empower the fourth industrial revolution. For instance, Blanco et al. [101] discusses a wireless actuator network used to create an IoT-based intelligent system, called smart actuators, which accomplishes a reduction in overall energy consumption at a given time by optimizing the functioning of devices and equipment in the IoT [102]. In addition, Llaria et al. [103] apply wireless actuators [104] for intelligent management techniques in a microgrid environment. Junior et al. [105] describe sensors used for management of energy generated by distributed renewable plants.

## 6. Conclusions and Future Directions

This survey presents a functionality study of significant electric actuators in different renewable applications and the current trends in usage. Traditional nonelectrical actuators are gradually being replaced with electric actuators. Primarily, four significant electric actuators’ motion classifications are found in the studied literature: rotary, linear, spherical, and seismic actuators. However, micro-level functionality analysis is necessary to find further energy optimization in renewable applications. Some of the studied conclusive vital points are listed below:

- Use of electric actuators are more advantageous in renewable applications as compared to non electric actuators by their quick response, low wight, precision in control, compact, clean, and more important IoT environment compatible.
- Rotary actuators are suitable wherever accuracy demand is lower but ample torque is needed to drive the load, as preferred in solar group tracking applications and solar furnace heliostat tracking applications. However, electrical cogging and mechanical dead zone nonlinearity increase with time.
- In discrete (step-by-step) tracking, increasing steps are uneconomical due to cumulative intermediate starting current being more than the continuous tracking current.
- In desert regions, net solar power improvement is approximately 3 W to 5 W. Further improvement is not achievable with available actuators; different actuators are required to remove the dust particles efficiently.
- Power consumption in BLDC is low (6 kWe) compared to a stepper motor (32 kWe) in the same capacity tracking application.
- Linear actuated generator with oscillating buoy gives more efficiency than a hydraulic drive, water turbine, or air turbine due to the reduction in power conversion loss in direct actuation.

Additionally, direct linear motion actuators without mechanical transmission elements and iron core free actuators reduce significant problems associated with rotary-to-linear actuators such as cogging, eddy current, heat, magnetic saturation, and lateral forces and hence play an important role nowadays. The spherical actuator is well-suited to solar tree applications due to less space requirement and requires two degrees of freedom. Overall, electric actuators have become more suitable and popular than convectional nonelectrical actuators in renewable applications due to their lower power consumption. Identifying different actuator control problems and challenges of electric actuators such as nonlinearities, saturation problems, and opportunities in optimization and their appropriate control strategies to overcome actuator-related issues for optimal use in renewable applications. The effectiveness of such control methods is part of a possible future work. Major performance parameters are discussed to facilitate actuator selection, which helps find significant parameter-related actuator selection. Next-generation, smart, IoT-based actuators will play an essential role in the energy sector and the chemical and automobile industries due to their two-way communication wireless technologies.

## Figures and Tables

**Table 1:** Energy consumption in continuous and step-by-step tracking [52].

Number of Steps	One-Day Energy Production (Wh)	One-Day Energy Consumption in Actuator (Wh)	One-Day Energy Gain (%)
0 (i.e., continuous)	898.46	12.82	39.42
8	893.27	13.32	38.21
6	891.73	12.99	38.02
4	887.32	12.92	37.34
2	868.63	12.88	34.41

**Table 2:** Revolution in application of solar furnace actuator installed at Font Romeu Odeillo.

Generation	Year	Actuator	Power Consumption in Actuator	Maintenance Frequency	Degree of Cleanliness
First generation	1968–1994	Hydraulic jacks	No available data	High (due to hydraulic leaks)	Very less
Second generation	1992–2014	Stepper motors with ball screw jacks	Up to 32 kWe	Moderate	Good
Third generation	2014 onwards	BLDC with ball screw jacks	Up to 6 kWe	Low	Good

**Table 3:** Summary of actuator power consumption in different applications.

Application	Axis/Tracking Nature	Actuator Type	Location	Generation Capacity	Actuator Power Consumption	Net Power Improvement	Simulation/ Experiment	Feature
Solar tracking	Single continuous	Linear [53]	Central Vietnam	250 W	2–8%	up to 30.3% (sunny), up to 15.2% (other)	Experiment	Grid connected solar plant
	Single continuous	Linear [12]	Hermosillo	No available data	No available data	No available data	Simulation	Cheaper solar array tracking
	Single continuous	Linear [48]	NA	NA	for +$45^{\circ }$ more than $-45^{\circ }$	NA	Simulation	Optimum actuator location
	Single continuous	Rotary [46]	Zimbabwe	24 W	No available data	up to 25%	Experiment	Affordable tracker
	Dual continuous	Linear [79]	Culiacán, Sinaloa	135.2 W	6.71 W	No available data	Experiment	Maintain power level
	Single, Intermittent (3-step)	Linear [51]	Irbid	0.4 W	Very less	around 91–94%	Experiment	Optimized 3 steps
	Dual, Intermittent	Rotary [50]	Bangladesh	NA	0.6 W	Up to 25.62% (compared to static)	Experiment	Low consumption in actuator
Solar panel cleaning	No tracking	linear piezoelectric [41]	No available data	NA	6.8 W	Increased by 1.29 W	Laboratory testing	Energy gain improvement
	No tracking	Piezoelectric [15]	California	No available data	No available data	up to 95%	Experiment	Creates vibration
	No tracking	Linear [80]	India	20 W	No available data	upto 3.35 w	Experiment	For street lights
	No tracking	Wobbling [70]	Egypt	130 W	No available data	up to 2.912 w after 6 weeks	Experiment	For desert regions
	No tracking	Geared motor [81]	Saudi Arabia	200 W	No available data	up to 4.78%	Experiment	For dry and desert regions
Solar furnace	Single continuous	Stepper motors with ball screw jacks [43]	France	1 MW	Up to 32 kWh	No available data	Experiment	Solar furnace with heliostat tracking
	Single continuous	BLDC with ball screw jack [43]	France	1 MW	Up to 6 kWh	No available data	Experiment	Solar furnace with heliostat tracking
Solar heating	NA	Rotary valve [14]	Portugal	NA	No available data	From 250 ∘C to 260 ∘C	Experiment	Only controlling flow
	NA	Rotary valve [63]	Spain	NA	No available data	From 190 ∘C to 245 ∘C	Simulation	Flow control

NA: Not applicable.

**Table 4:** Comparison of the wind turbine actuators.

Parameters	LAT Actuator [42]	Stepper-Motor-Based Actuator [82]
Power consumption	high	low
Complexity	low	medium
Weight	high	low
Reliability	high	low
Electronic control complexity	low	medium
Energy harvested at high speed	medium	high

**Table 5:** Efficiencies of different PTO.

Actuator and PTO Combination Type	Efficiency (%)
Hydraulic drive with oscillating body	65 [85]
Water turbine with oscillating body	85 [85]
Air turbine with OWCs	55 [85]
Mechanical drive with oscillating body	90 [85]
Piezoelectric device with oscillating body	very low [86]
Linear generator with oscillating buoy	95 [85], 90.03 [86]
Linear generator with OWCs	83.6 [87]

**Table 6:** Summary of Major Performance Parameters, Specifications, and Applications.

Types of Actuator	Major Performance Parameters/Specifications	Applications
Electric rotary actuator (AC motor) [13,60]	Shutter travel time: 0.5 s for opening and closing	Solar furnace shutter operating system
Piezoceramic actuators [15]	Frequency range: 400–5000 Hz	Solar array dust removal system
Electric rotary actuator (DC Motor) [47]	Motor speed and gear ratio	Solar tracker
Piezoelectric actuator [16]	DC voltage: 6 v, panel inclination angle	Solar panel cleaning
Relay [20]	Max. coil voltage, Max contact current capacity	Controlling greenhouse parameters
Electromagnetic clutch [20]	Coil voltage, clutch torque	Bio-gas stove
Induction servo motor (ISM) [21]	Operating voltage, operating speed (in sec/deg), torque (kg/cm or N/m)	Agricultural machines
Latch type solenoids [23]	Operating voltage: 12 V DC, Connection size one-eighth inch, Valve bore: 2.5 mm, Nominal power: 7 VA	Localized irrigation system
AC induction motor [46]	Gear ratio	Biomass grate boiler
Linear actuator [46,48,49,53]	Stroke length: 1005 mm, voltage: 12 V DC, speed: 4 mm/sec, force: 1500 N	Tracking applications
Rotary valve actuator [61]	Range of motion, Valve Stem Stroke Length, Actuation Time, Fail-safe	Solar heating system
Linear piezoelectric actuator [41]	Plates size: 10 mm × 10 mm × 1 mm, piezoelectric material: PZT-8H, operating frequency: 18.8 kHz and operating voltage: 100 $V_{o-p}$	Solar panel cleaning, astronautic and aeronautic applications
Limited-Angle Torque (LAT) actuator [42]	Threshold coil voltage, rotating angle $0^{\circ }$ to $90^{\circ }$	Over-speed protection of wind mill
Solenoid [94]	Stroke length: 10mm, Operating voltage: 24 V DC	For limited stroke on–off position.

**Table 7:** Comparison of electric actuators used in the various renewable applications.

Name of the Actuator	Advantages	Disadvantages	Limitations
Spherical actuator	Less space required Low power consumption Available in desired DOF Compact	No counterbalance Sensitive to vibration	Suitable for discrete tracking For moving large weight, latch is required for power saving Can be used for single panel tracking only
Rotary actuator	Suitable for group tracking applications	Backlash nonlinearity effect is more Quick response cannot obtained due to dead-zone nonlinearity	Cannot provide precision motion accuracy
Linear actuator	Backlash nonlinearity effect is less Compact than rotary actuator	Large space required in solar tree application	Space limitation for solar tree applications
Piezo-electric actuator	Light weight so no adverse effect on solar panel tracking system.	Surface vibration area is very less Complex and costly control circuitry	Need more than one actuator for effective vibration effect
LAT actuator	Automatic operation based on threshold voltage, no extra control required Low complex than rotary actuator	Power consumption is high than rotary actuator	Applicable only for up to 2.5kW domestic wind turbine speed control application
Solenoid linear valve actuator	Operate in pressurized fluid flow Fast response time Economic feasibility	Supply voltage deviations affects performance	Cannot obtain intermediate stage

## Data Availability

Not applicable.
